# Bristol and Bordeaux

**Published:** 1957-07

**Authors:** J. A. Cosh

**Affiliations:** Consultant Physician, Bath Clinical Area


### BRISTOL AND BORDEAUX

BY

J. A. COSH, M.D., B.CHIR., M.R.C.P.

*Consultant Physician, Bath Clinical Area*

For the past ten years relations between Bristol and Bordeaux have been fostered by the Bristol–Bordeaux association, which, among other things, links the schools and the universities in the two cities. The association's main function is the organization of the exchange of numbers of school-children for visits of two weeks or more each summer. Individual visitors are also exchanged between the universities, and recently lecturers have been exchanged between the faculties of medicine. In April 1956 I had the pleasure of visiting Bordeaux for a week, when, in return for two lectures given to medical and student audiences, my wife and I were warmly and hospitably received by members of the medical faculty in their hospitals and departments, and in their homes. In November last year we in Bristol were visited by Professor Massé, Professor of Surgery in Bordeaux. He gave us lectures on the aims and organization of the French blood-transfusion service, of which he is the regional head, and on the preservation of arterial grafts. Although his visit here was brief, we were able to show Professor Massé something of our hospitals, university and city, and the regional transfusion service.

### THE CITY OF BORDEAUX

Bordeaux is a city of almost the same size as Bristol, lying at a bend in the broad river Garonne some sixty miles from its entry into the Atlantic. It has been the natural capital of its region, Aquitaine, for many centuries, and the remains of a Roman amphitheatre within the city testify to its importance in Roman days. Our hosts reminded us with pride that Bordeaux was an English city for three hundred years, beginning in 1154 when it became an English possession on the marriage of Henry II with Eleanor of Aquitaine. The Bordelais of those days evidently flourished under this arrangement, appreciating their independence from the throne of France as well as their convenient distance from the throne of England. Trade grew between Bordeaux and England, largely through the port of Bristol, and the main commodity shipped was wine, for which Bordeaux was already famous. Both the cathedral and the university were founded in the days of English rule, and later Bordeaux became the centre of the court of the Black Prince; it was here that his son, subsequently Richard II, was born. The English were finally defeated in 1453, in the Hundred Years' War, after which Bordeaux became a French city. The city's greatest days were, perhaps, in the eighteenth century, and there are many fine boulevards, squares and buildings, notably the magnificent theatre, dating from this period. The main industrial activity of Bordeaux and its countryside is still the wine trade, now recovering from the effects of the war; we spent some pleasant and instructive hours in the famous wine-producing districts near Bordeaux—Médoc, Graves and Sauternes—seeing and tasting their products. We quickly realized, too, that Bordeaux has a well-deserved reputation for food as well as for wine. In touring the city we were interested to see the big new stadium and sports park, where the influence of Spain and the Basque country is shown by the occasional holding of a bull-fight or pelota match.

### HOSPITALS AND MEDICAL SERVICES

We visited a number of hospitals in and near Bordeaux. For the most part they are housed in old and undistinguished buildings, in which the processes of expansion and modernization are as painful and costly as they are in England. However, reconstruction is going ahead, if slowly. On the whole, laboratory equipment seemed at least equal to ours, although wards in the older buildings were often dreary. There was certainly no lack of talent, industry and ingenuity among the medical staff. My own particular interest was in the cardiac department, under the charge of Dr. Broustet. This was evidently not a show place, and was presented rather apologetically; nevertheless space was ample, laboratory equipment was in many ways better than our own, and I was impressed by the work on cardiac and pulmonary function being carried out by Dr. Bricaud. On one of my visits I watched a mitral valvotomy performed by Dr. Dubourg, and in the course of discussion found that they had done this operation nearly as often as we have in Bristol, and that our experiences with it are very similar. The chest hospital and sanatorium newly built a few miles from Bordeaux was magnificent, and lavishly equipped. Here again the talent and character of the medical staff were impressive, and I was interested to see a colour film of bronchoscopic appearances in various conditions made by Dr. Fréour.

Medical care in France is based upon a system of compulsory insurance which does not go as far as ours in England. The patient receives attention from his doctor, from a specialist of his own choice, or in hospital, and the cost is defrayed to a limited extent by the insurance. Those who can, prefer to enter nursing homes or private clinics, where the fees, of course, may be far above the sum provided by the insurance. Nearly all doctors rely upon private practice for their income, for the remuneration from insurance and hospital work is low, and the cost of living is high. Consequently the general practitioner does not have the standing that he does in England, particularly in the French cities. The majority of doctors obviously prefer to specialize when they can, and not uncommonly help to establish clinics or hospitals which are based upon private investment, or a mixture of private and communal investment.

We found on all sides a most lively interest in the National Health Service, tempered by a polite scepticism. Vigorous discussions ensued on the relationships between the universities and the Health Service: strong views were held for and against the desirability of whole-time posts for medical staff in teaching hospitals. Conservative French opinion is deeply attached to the ideals of independence and private enterprise, while the younger generation, being less committed to the *clientèle privé* as the basis of existence, is more prepared to accept some form of partly-salaried health service, or whole-time posts in certain hospitals, However, conthe main flict of opinion seems yet to come.

### THE MEDICAL FACULTY

The medical school at Bordeaux is not only several hundred years older than ours, but is also very much bigger. This is mainly because French medical schools and universities, which are financed and controlled by the Ministry of Education, are bound to accept all students capable of entering, without limitation of numbers. Students fees are a fraction of ours, and are virtually only token payments. The annual entry of students averages about 300, compared with our 60, resulting in phenomenal difficulties in teaching, particularly in laboratory work and clinical training. The medical course lasts six years. After spending the first year on science subjects, students enter the Faculty of Medicine, and throughout the second to sixth years it is the custom to spend mornings at the hospitals and afternoons in the faculty at lectures or in the laboratory. There are annual examinations, the main clinical examinations coming after the fifth year, and the fail rate in the earlier years may be as high as 20–30 per cent. If all goes well, however, the student submits a thesis after his sixth year, and acceptance of this brings him the degree of M.D. and the right to practise.

For the rank and file of students, clinical training and practical experience must necessarily be very limited. However, after the second, third and fourth years students compete by examination for some fifty positions each year as "externs". Externs work as clinical assistants in various departments in rotation in the mornings, continuing their studies in the afternoons; they are non-resident, and receive a small emolument. After the fifth year ten "interns" are chosen each year by examination, and they correspond to our resident house physicians and surgeons, and are paid some £50 per year. Some may serve a second or third year's internship, ultimately presenting their thesis for the degree of M.D. in the usual way. Subsequently these men will have the right to announce on their brass plates that they are "anciens interns des hôpitaux de Bordeaux".

A man wishing to go further in the teaching hospital will then compete by examination for the post of *chef de clinique* becoming a junior member of the university staff (and thus under the Ministry of Education) corresponding to our registrars and junior lecturers. A position on the staff of a non-teaching hospital, under the Ministry of Health, may be gained, again by competition as *médecin des hôpitaux* or *chirurgien des hôpitaux*. Further advancement in the teaching hospital means a final examination for the position of *professeur agrégé* at about the age of 40. Most, but not all, *professeurs agrégés* can hope to be elected to a full professorship some ten years later. None of these posts carry more than a nominal salary, so that every doctor above the level of intern has to take up private practice for a living, usually in a specialty. Higher, or specialist, degrees are uncommon in France, and the energies of ambitious doctors are evidently fully absorbed by the system of advancement by competitive examination.

THE NAVAL MEDICAL SCHOOL

At Bordeaux the annual entry of 300 students includes about 100 from the French naval medical school. This is the only one of its kind in France. and has its parallel in the army medical school in Lyons. A young man wishing to serve as a doctor in the French navy or oversea possessions may enrol in the navy after passing his *baccalaureat* at the age of 17 or 18. He then lives in the naval medical college in Bordeaux under a modified naval discipline, and is uniformed as a cadet. He is there given his initial naval training and his introductory science course, and, if he wishes, such interesting extras as parachute jumping. He then enters the Bordeaux medical school with the second year and follows the usual course through with them. After graduating he is, of course, contracted to serve for a number of years in the navy or oversea. A keen interest is taken in rowing (*canotage*) at the naval medical school, and it is their crews who have competed with Bristol University crews in the past.

CONCLUSION

While in Bordeaux I was assured by M. Sigalas, the dean of the medical faculty, that a warm welcome would be given to any of our staff or students visiting the hospitals or medical school there, and on his visit to Bristol Professor Massé endorsed this invitation. The exchange of visitors between Bristol and Bordeaux continues, and it is clear that in other spheres as well as in medicine, friendship and understanding between Britain and France will be strengthened by this link between our cities.

My thanks are due in particular to Dr. Broustet and to M. Loiseau, Professor of English, for their great kindness, and the trouble that they took to make our visit so enjoyable.

